# Comparative e-waste plastics biodegradation efficacy of monoculture *Pseudomonas aeruginosa* strain PE10 and bacterial consortium under *in situ* condition

**DOI:** 10.3389/fmicb.2023.1277186

**Published:** 2024-01-18

**Authors:** Prasenjit Debbarma, Deep Chandra Suyal, Saurabh Kumar, M. G. H. Zaidi, Reeta Goel

**Affiliations:** ^1^Department of Botany, Iswar Chandra Vidyasagar College, Belonia, Tripura, India; ^2^Department of Science, Vidyadayini Institute of Science, Management, and Technology, Bhopal, India; ^3^Division of Crop Research, ICAR-Research Complex for Eastern Region, Patna, Bihar, India; ^4^Department of Chemistry, College of Basic Sciences and Humanities, G. B. Pant University of Agriculture and Technology, Pantnagar, Uttarakhand, India; ^5^Department of Biotechnology, Institute of Applied Sciences and Humanities, GLA University, Mathura, UP, India

**Keywords:** e-waste, consortium, monoculture, biodegradation, bio-recycling

## Abstract

A significant amount of electronic obsoletes or electronic waste (e-waste) is being generated globally each year; of these, ~20% of obsolete electronic items have plastic components. Current remediation practices for e-waste have several setbacks due to its negative impact on the environment, agro-ecosystem, and human health. Therefore, comparative biodegradation studies of e-waste plastics by monoculture *Pseudomonas aeruginosa* strain PE10 and bacterial consortium consisting of *Achromobacter insolitus* strain PE2 (MF943156), *Acinetobacter nosocomialis* strain PE5 (MF943157), *Pseudomonas lalkuanensis* PE8 (CP043311), and *Stenotrophomonas pavanii* strain PE15 (MF943160) were carried out *in situ*. Biological treatment of e-waste with these candidates in soil ecosystems has been analyzed through diversified analytical techniques such as Fourier transform infrared spectroscopy (FTIR), thermogravimetric-derivative thermogravimetry-differential thermal analysis (TG-DTG-DTA), and scanning electron microscopy (SEM). Both *P. aeruginosa* strain PE10 and the bacterial consortium have a tremendous ability to accelerate the biodegradation process in the natural environment. However, FTIR analysis implied that the monoculture had better efficacy than the consortium, and it was consistent until the incubation period used for the study. Some polymeric bonds such as ν C=C and δ C-H were completely removed, and ν C=C ring stretching, ν_asym_ C–O–C, ν_sym_ C–H, etc. were introduced by strain PE10. Furthermore, thermal analysis results validated the structural deterioration of e-waste as the treated samples showed nearly two-fold weight loss (*W*_L_; 6.8%) than the untreated control (3.1%) at comparatively lower temperatures. SEM images provided the details of surface disintegrations. Conclusively, individual monoculture *P. aeruginosa* strain PE10 could be explored for e-waste bio-recycling in agricultural soil ecosystems thereby reducing the cost, time, and management of bioformulation in addition to hazardous pollutant reduction.

## Graphical Abstract

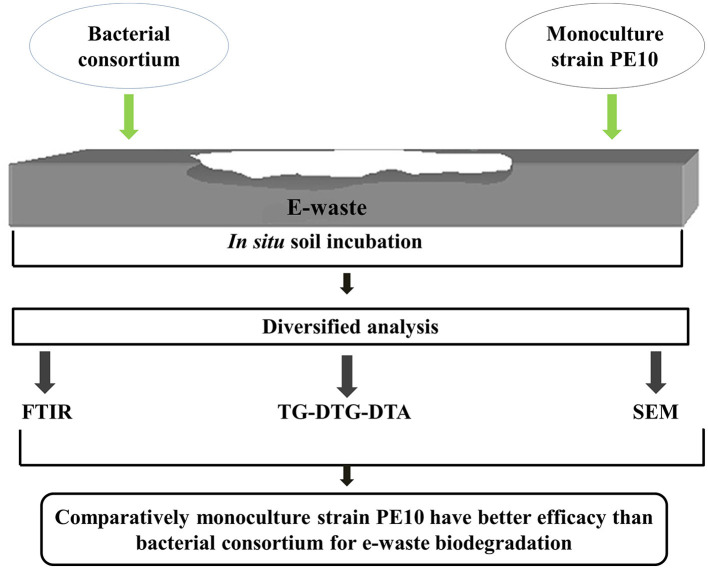

## 1 Introduction

Electronic waste (e-waste) possesses various hazardous and non-hazardous substances, and thus, it is a complex waste stream. Therefore, the challenge involved in the appropriate management of e-waste is crucial to sustaining our ecosystem, livelihood, and environment. The sustainable approach comprises a challenging task to the digital societies which would further necessitate organized efforts to deal with e-waste. Existing conventional practices have failed to manage these huge electronic obsoletes sustainably, and therefore, the waste is growing exponentially around the world (Forti et al., 2020). Based on chemical composition, e-waste contains mainly metals (60%), plastics and their blends (30%), and other harmful materials (10%) (Gaidajis et al., 2010). However, this composition is so complex that it varies with different electronic items of different categories. Moreover, different types of thermoplastics such as polyvinyl chloride (PVC), polyurethane (PU), polystyrene (PS), high-impact polystyrene (HIPS), and acrylonitrile–butadiene–styrene (ABS) are present in e-waste (Mohan et al., 2016; Sekhar et al., 2016; Debbarma et al., 2018). Hazardous substances such as various additives (organic and inorganic) and fillers are used in plastics to enhance the material properties (Morf et al., 2007; Erickson and Kaley, 2011).

All these materials of e-waste release very harmful gases and inert chemicals, viz., CFCs, di-oxides, and furans, when they are incinerated. These compounds are potentially carcinogenic to humans, and thus, it is a serious concern. Furthermore, the landfilling of e-waste can deliver these dangerous materials to the groundwater, and they accumulate as leachates. During the recycling processes, harmful particles containing flame retardants and heavy metals are also discharged into the atmosphere (Kiddee et al., 2013). Therefore, the accumulation of e-waste in the environment is a major issue of the current era, and the management of this waste is thus a daunting task that needs to be tackled in an eco-friendly manner. Traditional methods because of their disadvantages have fuelled the use of biological tools to recover the precious metals present in e-waste and to promote the studies of bioleaching and biodegradation processes.

In the past decades, microbial leaching and bio-hydrometallurgical techniques have been exploited for the recovery of base and precious metal ions from e-waste (Brandl et al., 2001; Shah et al., 2015). Therefore, remediation of other toxic materials such as plastics present in e-waste is incomplete. Literature focusing on the direct biodegradation of complex e-waste is also rare. Recently, a group of researchers addressed the problem and studied the biodegradation of e-plastic. Potential isolates, viz., *Alcaligenes* sp., *Enterobacter* sp., *Citrobacter sedlakii* and *Brevundimonas diminuta*, and *Pseudomonas* and *Bacillus* strains are found to be able to degrade high-impact polystyrene (HIPS) present in e-plastic (Mohan et al., 2016; Sekhar et al., 2016). Zhu et al. (2021) studied the biodegradation of e-plastics, namely, polyurethane (PU), polystyrene (PS), and acrylonitrile–butadiene–styrene (ABS) present in e-waste by a wax moth's (Galleria mellonella) gut microbes, namely, *Enterococcus* and *Enterobacter*, respectively. However, in all those cases, the biodegradation studies were targeted only for a single polymer at a time. Moreover, previous studies have not carried out *in situ* biodegradation experiments; therefore, in a previous study by the author group, five new potential e-waste degrading bacteria were identified which were originally isolated from polluted soil and found to be very promising for bioremediation (Debbarma et al., 2018; Thorat et al., 2020). In the above context, the present study is conducted to compare the efficacy of monoculture *Pseudomonas aeruginosa* strain PE10 with the bacterial consortium for e-waste biodegradation under *in situ* conditions. Therefore, this investigation would further unravel the anomaly between the use of monoculture and consortium for effective large-scale biodegradation of synthetic polymeric e-waste and their exploration in waste management. This study may have important implications for e-waste bio-recycling and sustainable ways to tackle the e-waste crisis at present and in the coming decades.

## 2 Materials and methods

### 2.1 Materials

Randomly discarded computer keyboards were collected, and plastic materials mainly keycaps were sorted out from the rest of the wastes, viz., printed circuit boards (PCBs), metals, glasses, and wires. Thereafter, sorted e-waste plastics are grounded under “Wiley^®^ Mill” and sieved (~5 mm) through to collect e-waste granules. Then, e-waste granules are washed with 70% EtOH for 15 min. and dried at 50 ± 1°C (dry oven) for 1 h before using those e-waste granules as primary carbon sources. Soapstone (HiMedia, India) used as carrier material for bioformulation consists of talcum powder; steatite; talc, fine powder; and hydrous magnesium silicate.

### 2.2 Characterization of e-waste by FTIR spectroscopy

Prior to conducting the experiment, milled e-waste regarded as pure e-waste was subjected to an analysis of its chemical composition for characterization using a Fourier transform infrared spectroscopy (FTIR) spectrophotometer (Perkin Elmer version 10.03.06). The characteristics of FTIR absorbance are illustrated as wave numbers (cm^−1^) in the range of 4,000–450 cm^−1^.

### 2.3 *Pseudomonas aeruginosa* strain PE10 and bacterial consortium

The cultures of *P. aeruginosa* strain PE10 (NCBI accession no. MF943159) and consortium comprising of *Achromobacter insolitus* strain PE2 (MF943156), *Acinetobacter nosocomialis* strain PE5 (MF943157), *Pseudomonas lalkuanensis* PE8 (CP043311), and *Stenotrophomonas pavanii* strain PE15 (MF943160) were revived from 50% glycerol stocks (stored in −80°C at Departmental Culture Collection, Department of Microbiology, College of Basic Sciences and Humanities, GBPUAT, Pantnagar) by inoculating into 5.0 ml nutrient broth test tubes and incubated at pH (7 ± 0.2) and temperature (35 ± 1°C) for 24 h. Furthermore, aliquots of 500 μl overnight culture were used to inoculate into 10 ml nutrient broth and incubated for another 4 h at ambient growth conditions until an optical density (OD) of 0.6 was attained at 600 nm (OD_600_) to obtain mid-log phase active culture. All the used cultures in this study were originally isolated from the Net House Experimental Pit, Pantnagar, and from a dump yard of the Century Pulp and Paper Mill, Lalkuan, Uttarakhand, India (Debbarma et al., 2018; Thorat et al., 2020).

### 2.4 Preparation of bioformulations and shelf-life determination

The active consortium (800 ml) was divided into 16 parts, 50 ml each in centrifuge tubes and spun at 5,000 rpm for 10 min to separate the cells using a Sigma 3–16K centrifuge. Later, the supernatant was partially decanted, and then the tubes were vortexed for 15 min. Then, 5 g soapstone was weighed and added properly to each tube with pellets under sterile conditions. The tubes were vortexed again for a homogenous mixing of talc with the bacterial suspension. With a sterile spatula, the mixture was then emptied into a glass dish and kept at room temperature (28 ± 1°C) aseptically for drying the mixture. Later, the viability of bacterial strains in the formulation was ascertained according to a previous study (Goel et al., 2015) (Supplementary Text 1).

### 2.5 *In situ* efficacy studies

#### 2.5.1 In situ incubation of e-waste

Topsoil was dug from the Crop Research Center (CRC) at Pantnagar, India, and half-filled into 60 cm × 30 cm × 30 cm (length × width × depth) experimental net house pits. First, the soil was mashed manually and 15 g of e-waste granules were mixed with mashed soil for the treatments. Then, 200 g of prepared active bioformulation of the consortium was added to the soil of the treatment pits which was then incubated under natural conditions. Furthermore, autoclaved distilled water was sprinkled at regular intervals of 2–3 days to maintain the moisture content of the soil. Pit cleaning and aeration conditions were maintained by shoveling the soil at regular intervals of 15 days. Keeping the point of shelf-life of consortium in bioformulation, an active bioformulation was added in the pit at regular intervals of 60 days. The biodegradation study of the treatments, i.e., (a) soil + e-waste + monoculture *P. aeruginosa* strain PE10 and (b) soil + e-waste + consortium, was performed with respective positive (soil + e-waste) control. The study was carried out for a period of 6 months.

#### 2.5.2 Recovery of biodegraded samples

The treated e-waste samples from the soil pits (i.e., positive and treatment pits) were carefully recovered after 3 and 6 months of incubation and collected in sterile Whirl-Pak™ sample bags with the help of trowel/khurpi and sieved after the incubation period. The biodegraded samples were washed and surface-sterilized with 70% EtOH for 10 min and subsequently vortexed vigorously followed by drying at 50 ± 1°C (dry oven) for 1 h to evaporate leftover liquid residues. After washing with EtOH, the collected e-waste samples were added to 15 ml centrifuge tubes containing 10 ml of millipore water, and the tubes were then centrifuged at 5,000 rpm at 4°C for 10 min to remove the remaining soil particles and microbial biomass. Finally, the supernatant was carefully removed, and the leftover water was evaporated by placing the residues in an oven at 50 ± 1°C (dry oven) for 24 h.

#### 2.5.3 Comparative analysis of treated samples

##### 2.5.3.1 FTIR spectroscopy

The standards and programming of the spectrophotometer were maintained as same as mentioned in Section 2.2, wherein ν and δ are used to represent the stretching and bending vibrations, respectively.

##### 2.5.3.2 Simultaneous TG-DTG-DTA

The study of simultaneous thermogravimetric-derivative thermogravimetry-differential thermal analysis (TG-DTG-DTA) was performed for e-waste treated with monoculture *P. aeruginosa* strain PE10 and bacterial consortium using untreated e-waste control as a reference. This experiment was carried out to compare the thermal stability of biodegraded e-waste on an EXSTAR (SII 6300 EXSTAR) thermal analyzer under a nitrogen atmosphere at 200 ml/min programmed at 35°C to 800°C temperature range with a heating rate of 5°C/min on a platinum sample pan.

##### 2.5.3.3 SEM

Scanning electron microscopy (SEM) was performed to study the surface morphology of both treated samples and positive control samples. For this, the samples were thoroughly washed with 70% EtOH for 10 min and dried properly using a desiccator for 24 h under vacuum. Later, the samples were metalized with gold particles and observed under SEM (JEOL JSM-6610 LV) at 8.00 kV EHT with a magnification of 400×.

## 3 Results and discussion

### 3.1 Structural characterization of e-waste

**Figure 1:**
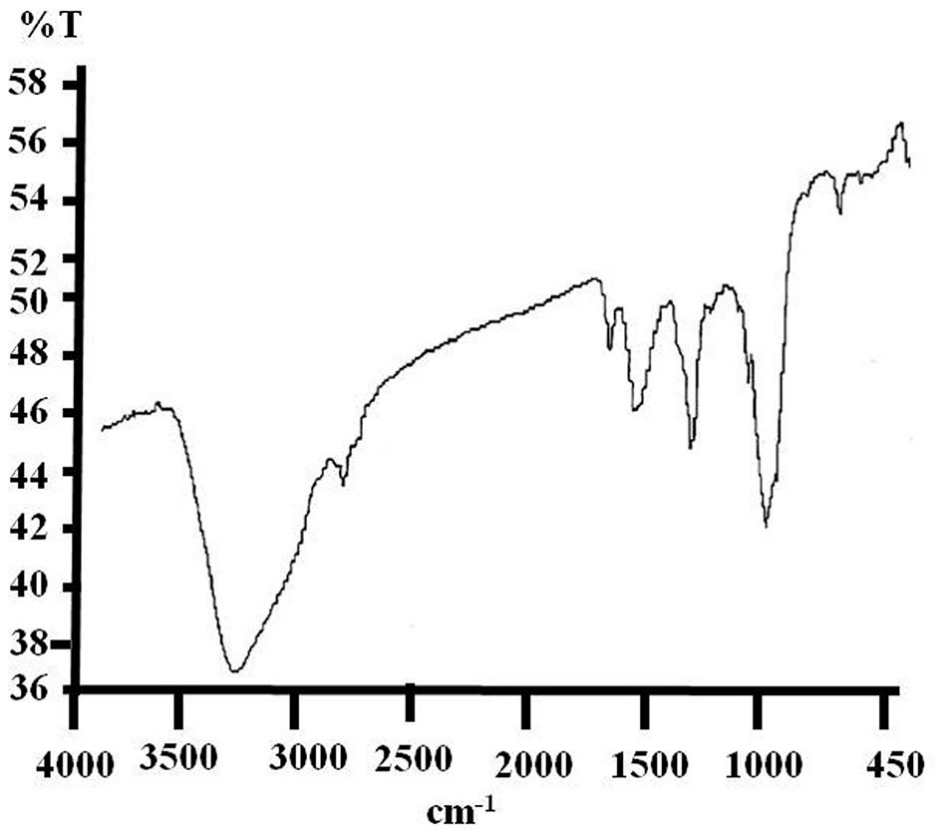
Charactersitic FTIR spectrum of pure e-waste showing the absorbtion peaks corresponds to its base polymers.

The analytical results of the e-waste FTIR spectrum corresponding to their polymers are summarized in Figure 1 (Supplementary Table 1). During the investigation, symmetrical and asymmetrical absorptions were observed for some bonds, and they are symbolized by “_asym_” and “_sym_,” respectively. Pure e-waste has shown the common characteristic wave numbers (KBr, cm^−1^) of O–H (3,390.49), _asym_ C–H (2,922.8), C=O (1,755.21), C=C (1,645.87), C–H (1,402.48), C–O (1,155.8), _sym_ C–O–C (1,069.02), and C–Cl (759.38), respectively.

The interpretation of the results identified typical spectra of base polymers which are acrylonitrile–butadiene–styrene (ABS) and high-impact polystyrene (HIPS) as well as some minerals. The FTIR spectrum of pure e-waste has shown evidence of the presence of acrylonitrile at a wavelength of 2,922.8 cm^−1^ corresponding to C-H bonds. Acrylonitrile can be present in PC/ABS blends due to the carbonyl (C=O) peak of the polycarbonate functional group observed at 1,755.21 cm^−1^ (Arnold et al., 2010). The absorbance bands observed at 1,645.87 and 1,402.48 cm^−1^ correspond to the benzene rings from the HIPS and C-H bonds which were used as reference peaks for the butadiene peak, respectively (Sekhar et al., 2016). Characteristic peaks related to the para aryloxy group and fillers at 1,155.8 and 1,069.02 cm^−1^, respectively, were also found (Vazquez and Barbosa, 2016). The peak at 759.38 cm^−1^ is characteristic of the presence of an aromatic ring or substituted phenyl ring. The presence of hydroxyl groups is identified by the peak which absorbs at 3,390.49 cm^−1^ wave number (Tiganis et al., 2002).

Therefore, in addition to ABS and HIPS, this absorbance indicated the possible presence of polymers such as polystyrene (PS), styrene-acrylonitrile (SAN), polycarbonate (PC), blends of polycarbonate (PC)/ABS, and blends of HIPS/poly(p-phenylene oxide) (PPO) as discussed by the abovementioned research groups. These varieties of polymers were found to be present in e-plastics which have good properties such as high-temperature resistance, mechanical strength, chemical stability, flame retardancy, rigidity, impact strength, and creep resistance (Beigbeder et al., 2013).

### 3.2 Bioformulations and its viability

Viability observations of monoculture and consortium confirm that cells present in the formulation were active even after 70 days of storage. The growth of both monoculture and consortium was slightly variable after 2 days of storage in terms of CFU ml^−1^, and a percentage (%) survival decrease was calculated. At 70 days, a percentage survival decrease rate for bacterial monoculture and the consortium was 4.84 and 3.88%, respectively, which suggests that bioformulations have considerable shelf-life longevity that allows the formulation to be used as a suitable carrier with active monoculture strain and consortium for *in situ* efficacy experimentation (Table 1).

**Table 1:** Viability of bio-formulations under ambient conditions during storage period.

**Bacterial agents**	**Dilution factor**	**CFU/ml at subsequent time intervals (days)**							
		**2nd**	**4th**	**11th**	**18th**	**25th**	**40th**	**55th**	**70th**
**Consortium**	10^7^	284 (±2)	283 (±2)	279 (±2)	280 (±2)	278 (±2)	275 (±2)	274 (±2)	273 (±2)
% survival decrease		0%	0.36%	1.77%	1.41%	2.12%	3.17%	3.53%	3.88%
**Monoculture strain PE10**	10^7^	165 (±2)	165 (±2)	164 (±2)	161 (±2)	163 (±2)	160 (±2)	159 (±2)	157 (±2)
% survival decrease		0%	0%	0.60%	2.42%	1.21%	3.00%	3.63%	4.84%

Each value is the mean of three replicates. Values in (±) indicate standard error.

The selection of the type of formulation developed and carriers used is dependent on the nature of active cells and the factors related to the site of application. Many other bioformulations have been reported in previous studies such as *Pseudomonas fluorescence, Rhizobacteria, Bacillus*, and *Pseudomonas oryzae* for the treatment of damping-off of cotton seeds and enhancement of induced systemic resistance, as a fertilizer and plant growth promoters (Bharathi et al., 2004; Mishra and Arora, 2016).

### 3.3 Efficacy analysis through diversified analytical techniques

During the soil incubation period under natural conditions, biodegraded samples from each experimental pit were recovered at 3- and 6-month intervals and subjected to qualitative analysis with reference to untreated control, respectively. Diversified analytical techniques, viz., FTIR, TG-DTG-DTA, and SEM analysis, were exploited for spectral, thermal, and morphological changes, respectively.

#### 3.3.1 FTIR spectra of biodegraded e-waste

**Figure 2:**
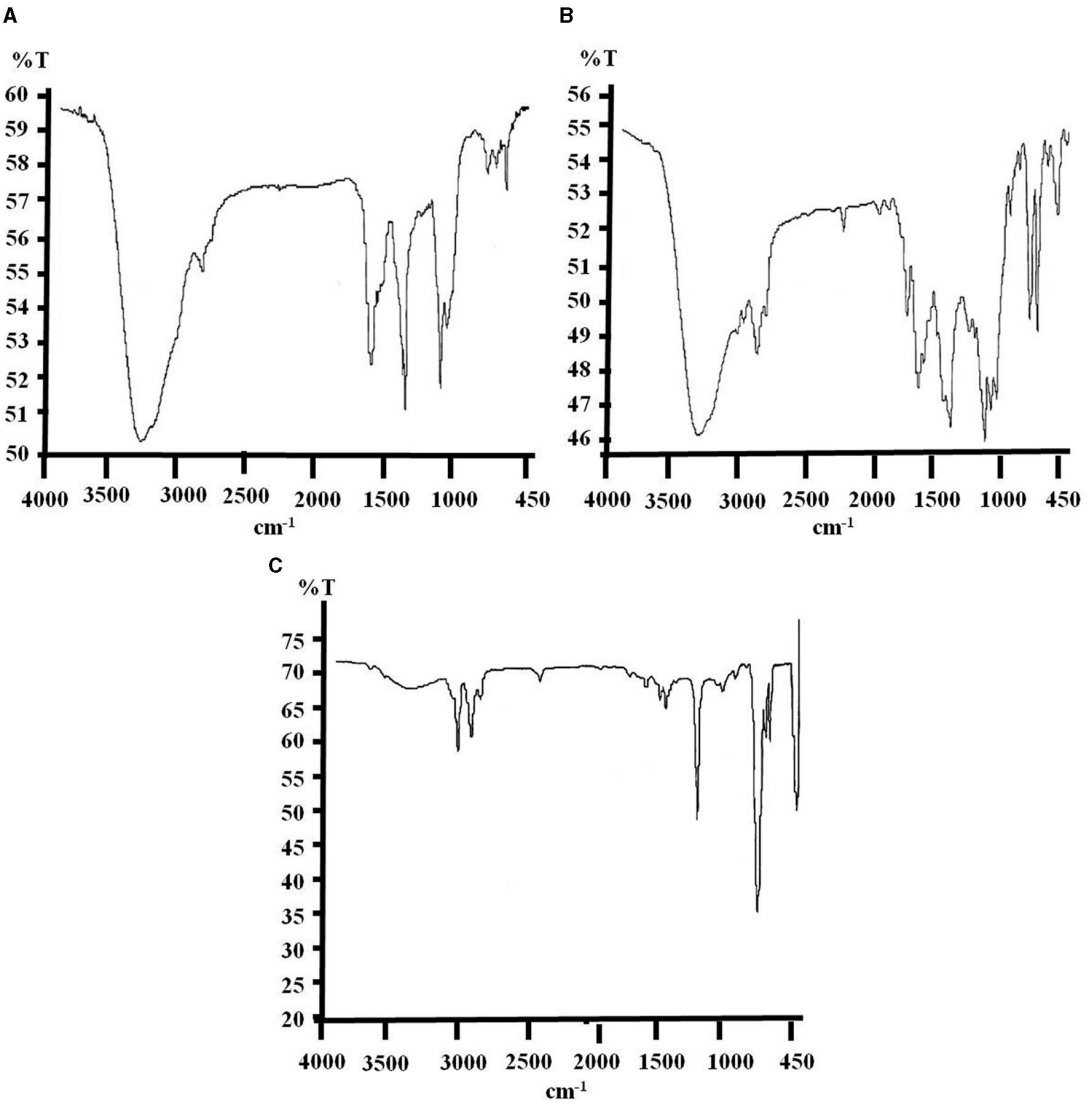
FTIR spectra of biodegraded e-waste samples, where spectrum **(A)** represent untreated control showing the absorption peaks with minor differences from pure e-waste spectrum due to environmental factors, after 3 months of soil incubation and spectrum **(B, C)** correspond to consortium and strain PE10 treated samples, respectively depicting significant changes and differences in absorption peaks at the same time of incubation period.

**Figure 3:**
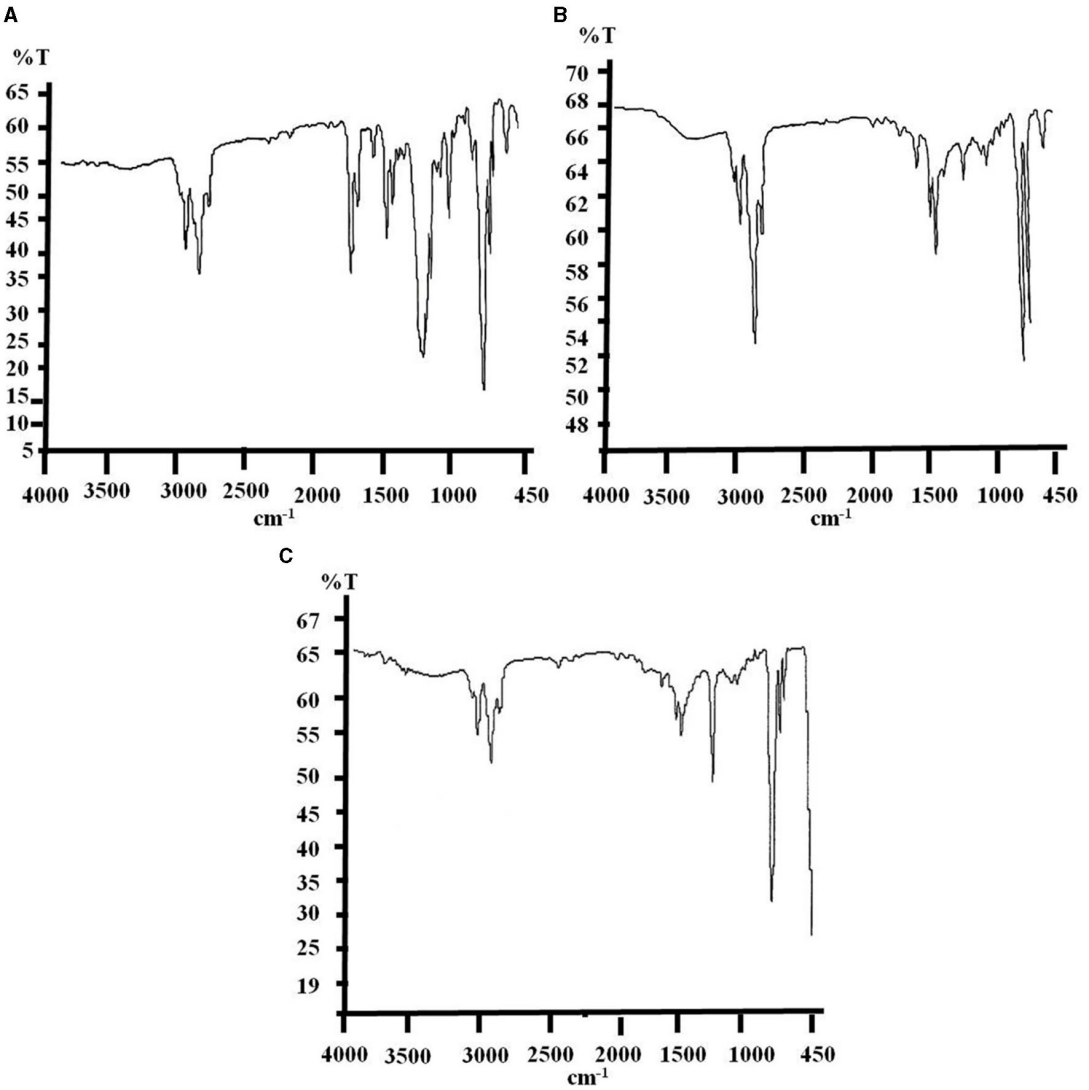
Final FTIR spectra of biodegraded e-waste samples after 6 months of soil incubation, where spectrum **(A)** portraying for untreated control with some changes in absorption peaks from last 3 months sample and spectrum **(B, C)** represents consortium and strain PE10 treated samples, respectively illustrating tremendous alterations in absorption peaks viz. removal, addition and shifting of wave numbers (cm^−1^) of peaks signifying the comparative biodegradation efficacy.

FTIR spectra for the relative functional potential of used monoculture and bacterial consortium toward the degradation of e-waste have been reflected through the changes in the wave numbers (cm^−1^) as well as the addition and deletion of functional groups and chemical bonds in the structure compared to untreated control. The changes in the chemical structural compositions of biodegraded e-waste are shown in Figures 2, 3 and Supplementary Tables 2, 3. Analysis of biodegraded samples has revealed variable peaks corresponding to diverse bond stretching and bending vibrations.

Untreated e-waste samples recovered from a soil bed after 3 months illustrated the wave numbers (cm^−1^) of ν OH (3,391.65), ν_asym_ C–H (2,923.58), δ C=O (1,730.09), ν C=C (1,641.54), δ C–H (1,386.95), ν C–O (1,117.49), ν C–Cl (758.79), and δ =C–H (701.06; Figure 2A and Supplementary Table 2). During this period of incubation under natural conditions, few changes in chemical structure have been noticed comparing the characteristic wave numbers of pure e-waste, i.e., the absolute deletion of ν_sym_ C–O–C peak and formation of a completely new peak that corresponds to δ =C–H (701.06 cm^−1^) bond. These changes may be attributed to the environmental conditions (temperature, light, heat, pressure, etc.) and also due to the unsterilized soil used in the study (Raghuwanshi et al., 2018). Comparison of e-waste exposed under bacterial consortium has shown remarkable changes in FTIR spectra such as the introduction of a new group aromatic δ =C–H corresponding to 618.41 cm^−1^. Total removal of ν_asym_ C–H, δ C=O, ν C–O, ν_sym_ C–O–C, and ν C–Cl from the structural composition directly reflects the action and efficacy of consortium for degradation of e-waste under soil ecosystem (Figure 2B). The effect of biodegradation on e-waste by monoculture *P. aeruginosa* strain PE10 is clearly seen in Figure 2C. This bacterium could degrade the e-waste just as the used consortium. The FTIR spectrum of PE10-treated e-waste elucidated four new peak characteristic to δ N–H (1,600.96 cm^−1^), ν C=C (1,450.99–1,492.80 cm^−1^) ring stretching, ν_asym_ C–O–C (1,215.11 cm^−1^), and ν_sym_ C–H (928.74 cm^−1^), respectively, as compared to the consortium-treated samples and the untreated control. The absolute removal of ν C=C, δ C–H, and ν C–O (Supplementary Table 2) groups from the polymeric backbone suggested that these changes are clearly attributed to the effect of strain PE10. Therefore, the 3-month *in situ* treatment result evidently suggests that both *P. aeruginosa* strain PE10 and bacterial consortium have remarkable efficacy for e-waste degradation under identical situations.

Furthermore, the final sample was recovered and collected from the soil bed after the completion of the incubation period, i.e., 6 months. FTIR absorptions of the untreated control samples showed additional peaks of ν C=C ring stretching and δ =C–H corresponding to the wave numbers (cm^−1^) of 1,452.18–1,502.65 and 669.26–701.42 cm^−1^, respectively. This sample also showed complete deletion of ν C=C and δ C–H functional groups as compared to pure e-waste (Figure 3A and Supplementary Table 3). A similar trend of changes in the spectrum was also observed after the 3-month soil incubation which could be attributed to environmental factors. However, the bacterial consortium-treated samples have depicted more significant degradation as the complete removal of functional groups such as δ C=O, ν C–O, and ν_sym_ C–O–C was more prominent for the consortium used in this study (Figure 3B). Nevertheless, a significant shift in the absorption frequencies such as the addition of δ N-H (1,600.69 cm^−1^) and ν_asym_ C–O–C (1,222.24 cm^−1^) was also observed in the samples treated with a consortium. Biodegradation with the consortium brought about significant shifts in the fingerprint region of the IR spectrum between 1,700 and 950 cm^−1^ of treated e-waste as compared to the control. Though, exposure of monoculture *P. aeruginosa* strain PE10 has induced remarkable changes in the spectra reflecting the complete degradation of δ C=O and ν C–O bonds from the structure of polymeric backbone and additional new absorption frequencies of ν N–H, δ N–H, and ν_asym_ C–O–C, i.e., at 2,402.35, 1,601.03, and 1,216.43 cm^−1^, respectively, were also observed (Figure 3C). Furthermore, reducing in the wave numbers of ν O–H, ν_asym_ C–H, ν C=C ring stretching and δ =C–H to 3,023.17, 2,852.67–2,924.19, 1,451.97–1,492.72, and 667.13–700.43 cm^−1^, respectively, were attributed by this culture unlikely to consortium and control. This result indicates that monoculture *P. aeruginosa* strain PE10 was rather more consistent in efficacy toward the progressive biodegradation of e-waste than the consortium. However, the bacterial consortium has shown advancement in its efficacy following the preceding sample analysis as the incubation period extends. Conclusively, comparative results of FTIR spectra analysis have clearly revealed that both bacterial consortium and monoculture *P. aeruginosa* strain PE10 have the potential to accelerate the biodegradation of e-waste under natural conditions. Therefore, to acquire further evidence on the organic degradation of the samples, simultaneous TG-DTG-DTA analysis was performed.

#### 3.3.2 Comparative thermal analysis

Biodegradation of e-waste particularly its organic fraction would reduce its thermal stability due to the composition changes after *in situ* treatment. Thermogravimetric analysis accurately determines the percentage weight loss (% *W*_L_) of the samples in accordance with programmed temperature and time interval conditions. Thermograms of the treated samples are portrayed in Table 2 and Supplementary Figures 1, 2 with reference to untreated control.

**Table 2:** Simultaneous thermal analysis of biodegraded e-waste under *in situ* conditions by bacterial consortium and strain PE10 with reference to the control after 3 months of soil incubations.

**Samples**	**%** ***W***_**L**_		**DTG**		**DTA**	
	**TG onset**	**TG endset**	*T*_max_ **(**°**C)**	**Rate (mg/**°**C)** × **10**^−3^	*T*_max_ **(**°**C)**	Δ*H*_f_ **(kcal/mole)** × **10**^−3^
Untreated control	7.2 (339°C**)**	96.0 (507°C)	419	15.8	422	3.8
Consortium treated e-waste	10.0 (321°C)	89.9 (660°C)	411	17.4	411	4.9
PE10 treated e-waste	6.9 (300°C)	84.4 (471°C)	409	24.7	412	5.1

The thermal analysis of 3-month samples indicated that the TG onset temperature 339°C with 7.2% weight loss (*W*_L_) of untreated soil control was much higher than bacterial consortium (321°C with 10.0% *W*_L_) and strain PE10 (300°C with 6.9% *W*_L_), respectively (Table 3). The percentage weight loss during TG onset clearly reveals the biodegradation of e-waste by the consortium, where the treated samples showed 10.0% *W*_L_ as compared with the untreated control which exhibited a weight loss of 7.2%, whereas *P. aeruginosa* strain PE10-treated sample has shown 6.9% *W*_L_ which was lower than both the untreated and consortium-treated samples; however, remarkably, reduced TG onset temperature shows the potential efficacy of this bacterium within this period of incubation. Moreover, the DTG peak of *P. aeruginosa* strain PE10 was observed at the lowest temperature of 409°C with the highest decomposition rate of 24.7 × 10^−3^ mg/°C among other samples, and the DTA peak was detected at 412°C with heat of fusion (Δ*H*_f_) 5.1 × 10^−3^ kcal/mole compared with other treated and untreated samples as seen in Table 3 and Supplementary Figure 1. Nonetheless, as the decomposition of the sample progresses under thermal influences, bacterial consortium-treated e-waste sample also showed considerable lower temperatures of the DTG peak at 411°C with rate of decomposition at 17.4 × 10^−3^ mg/°C and the DTA peak at 411°C with Δ*H*_f_ 4.9 × 10^−3^ kcal/mole in comparison with the control, where sample decomposition rate was at the lowest at 15.8 × 10^−3^ mg/°C with an elevated temperature of 419°C and the heat of fusion (Δ*H*_f_) was 3.8 × 10^−3^ kcal/mole at 422°C. Therefore, these findings were at par with FTIR results and thus further validated the significant efficacy of *P. aeruginosa* strain PE10 and bacterial consortium for e-waste biodegradation within an undistinguishable *in situ* environment.

**Table 3:** Simultaneous thermal analysis of biodegraded e-waste under *in situ* conditions by bacterial consortium and strain PE10 with reference to the control after 6 months of soil incubation.

**Samples**	**%** ***W***_**L**_		**DTG**		**DTA**	
	**TG onset**	**TG endset**	*T*_max_ **(**°**C)**	**Rate (mg/**°**C)** × **10**^−3^	*T*_max_ **(**°**C)**	Δ*H*_f_ **(kcal/mole)** × **10**^−3^
Untreated control	3.1 (300°C)	93.9 (418°C)	419	11.9	427	4.5
Consortium treated e-waste	6.8 (300°C)	90.1 (419°C)	412	18.5	418	5.3
PE10 treated e-waste	6.3 (300°C)	80.0 (432°C)	401	22.7	410	6.3

Furthermore, to establish the progressive nature of e-waste biodegradation by both treatments over a long period of incubation, thermal analysis of 6-month samples was characterized with reference to untreated control samples. As the soil incubation period was over, it was observed that all three samples have shown TG onset at 300°C with a significant amount of weight loss at this temperature, i.e., 3.1%, 6.8%, and 6.3% *W*_L_ for untreated control, consortium, and *P. aeruginosa* strain PE10, respectively (Table 2 and Supplementary Figures 2a–c). The decomposition of the treated samples at particular heat was remarkable as the percentage *W*_L_ was at least twice that of the control. In addition, the TG onset temperature requirement of 6-month samples was very much at minimum compared with TG onset temperatures of 3-month samples which suggested that the structural backbone of the polymers was disintegrated and the composition of the samples shattered by the influence of consortium to become brittle. Thus, the thermal stability of the biodegraded samples and TG onset temperature were reduced after the incubation period. Furthermore, these results are supported by DTG and DTA peak analyses, where the consortium-treated samples have shown considerably lower temperatures of DTG peak at 412°C with a rate of decomposition at 18.5 × 10^−3^ mg/°C, and the DTA peak was observed at 418°C with the heat of fusion (Δ*H*_f_) 5.3 × 10^−3^ kcal/mole (Supplementary Figure 2b). Comparatively, *P. aeruginosa* strain PE10-treated samples elucidated the lowest temperature of 401°C with the rate of decomposition 22.7 × 10^−3^ mg/°C along with DTA peak at 410°C showing the energy required for heat of fusion (Δ*H*_f_) 6.3 × 10^−3^ kcal/mole (Supplementary Figure 2c).

Conclusively, thermal analysis has clearly revealed the efficacy of bacterial consortium and *P. aeruginosa* strain PE10 which was determinately responsible for the progressive decomposition of biodegraded samples with much higher decomposition rate and increased weight loss at comparatively lower temperatures than the respective control after the long period of incubation. However, as seen during FTIR analysis, monoculture *P. aeruginosa* strain PE10 has proven to be consistent in efficacy, whereas the consortium was advancing in its efficacy as the incubation duration progressed. Changes in the thermal profiles of treated e-waste samples might be due to the action of bacterial enzymes with the functional groups present in the polymers, which, subsequently causes the alterations in chemical structure of the polymeric backbone as the result substantiated FTIR spectra. Thus, it was clear that both the consortium and *P. aeruginosa* strain PE10 could utilize e-waste polymer as their carbon and energy source when treated. Furthermore, the development of various DTG and DTA peaks was previously found and documented in the case of high-density polyethylene (HDPE) and low-density polyethylene (LDPE), polycarbonate, non-poronized and poronized LDPE (Kapri et al., 2010), epoxies and their silicone blends, and epoxy and cold-mix epoxy (CME) during the biodegradation studies (Shikha et al., 2015).

#### 3.3.3 SEM observations of recovered samples

Based on the comparative results obtained from FTIR and thermal analyses, it was confirmed that the treated e-waste was evidently degraded by the used consortium and monoculture strain PE10. Therefore, additional SEM micrographs were taken for conclusive evidence of e-waste biodegradation by the used bacterial agents under *in situ* conditions. Comparative analysis of the efficacy of *P. aeruginosa* strain PE10 and bacterial consortium on e-waste surface morphology at 3 and 6 months of soil incubation was apparently confirmed through SEM analysis.

**Figure 4:**
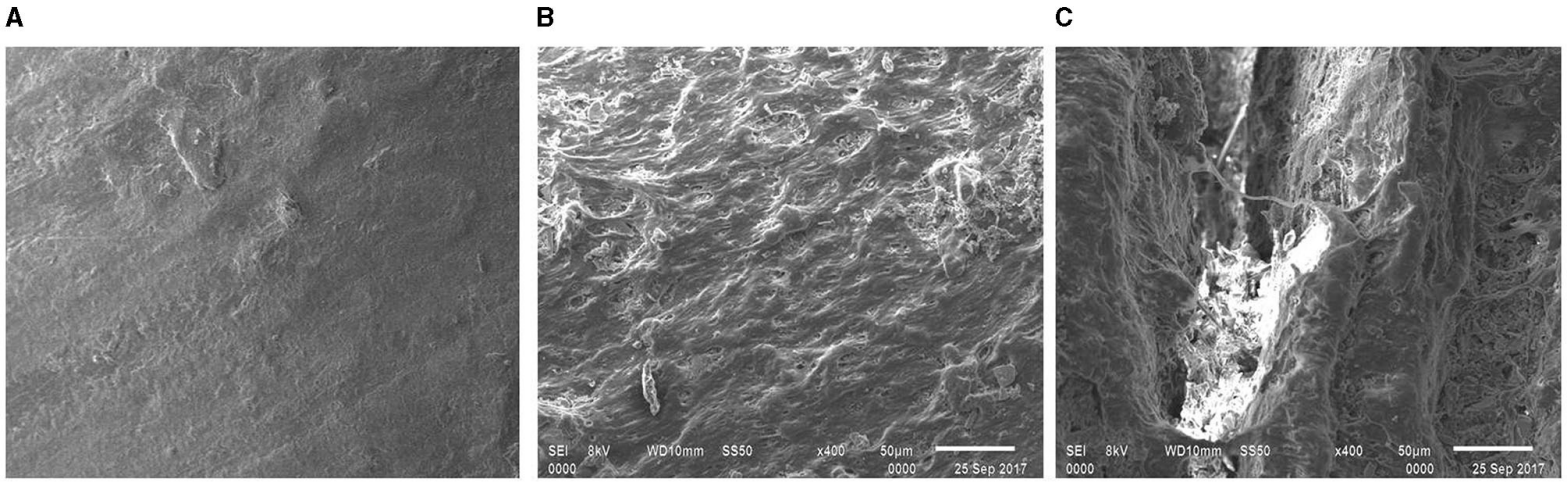
Comparative SEM micrograph of the e-waste recovered from untreated control **(A)**, bacterial consortium **(B)**, and strain PE10 **(C)** treated soils, respectively after 3 months of incubation. Scale bar = 50 μm; magnification = 400×.

**Figure 5:**
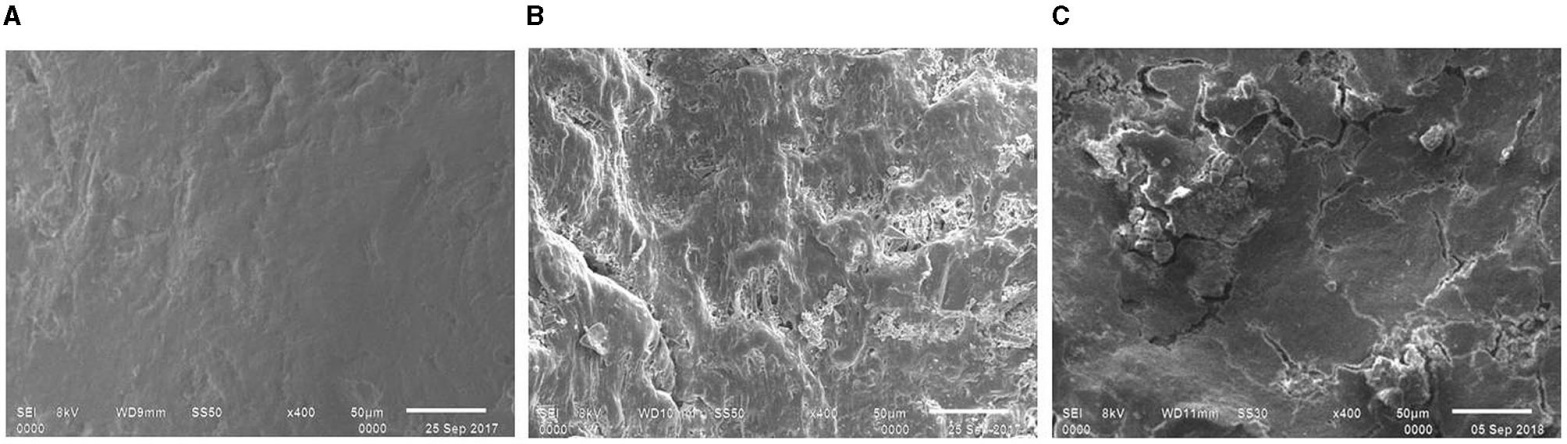
Comparative surface morphology of the e-waste recovered from untreated control **(A)**, bacterial consortium **(B)**, and strain PE10 **(C)** treated soils, respectively after 6 months of incubation. Scale bar = 50 μm; magnification = 400×.

During the incubation period, changes in e-waste surface morphology by the *P. aeruginosa* strain PE10 and the bacterial consortium were analyzed by taking untreated control samples as reference. The control samples from 3- and 6-month incubation revealed comparatively smooth and homogenous surface morphologies (Figures 4, 5A). However, the 3-month SEM image obtained from the treated (consortium and strain PE10) e-waste was visibly distinguishable from the untreated samples as the fissures and crumbles on the e-waste surface were extensive, showing major attributes, viz., well-resolved distortions, cracks, and formation of tiny cavities (Figures 4B, C). Furthermore, in the case of *P. aeruginosa* strain PE10-treated e-waste, the occurrence of fissures, heterogeneous morphology, fractures, and widened cracks was found to be remarkably predominant in comparison with the bacterial consortium.

Similarly, the surface resolutions, porosities, roughness, and cracks have been seen in the e-waste samples treated with the consortium and strain PE10 after 6 months of soil incubation (Figures 5B, C), whereas the control was comparable with that of the 3-month sample (Figure 5A). These heterogeneous surface morphologies on the surface of treated e-waste were obviously imparted after the exposure of bacterial agents which further substantiate the results of FTIR and thermal analyses. Thus, the SEM micrographs revealed the intensive surface deterioration of treated e-waste after soil incubation under natural conditions over successive periods of time. Similar biodegradation studies utilized SEM micrographs as a tool to provide evidence for the deterioration of the plastic film due to the action of the plastic degrading enzymes which demonstrate cavities and grooves formed on the plastic film, which directly reflected the extent of microbial colonization and degradation (Yoshida et al., 2016).

All the above analyses revealed that both the *P. aeruginosa* strain PE10 and the bacterial consortium have shown the capacity to degrade e-waste under *in situ* conditions with different contrasts. Therefore, further investigations such as proteogenomic study, degradation pathway prediction, and bacterial community analysis in the soil pit of the bacterial strains used may reveal more novel insights into the overall mechanism of e-waste biodegradation.

## 4 Conclusion

From this study, it can be concluded that both *P. aeruginosa* strain PE10 and bacterial consortium can potentially degrade e-waste under *in situ* conditions with their different levels of efficacy. The FTIR analysis of the biodegraded samples clearly proved that strain PE10 is as efficient as the bacterial consortium for the biodegradation of e-waste. It is also speculated that monoculture had consistency in its efficacy throughout the experimentation period, unlike the consortium which rather perpetuates its efficacy with the progression of incubation time.

Thermal analysis and SEM images of degraded samples further strongly substantiated these findings where monoculture-treated samples have shown thermal decaying at lowest temperatures with maximum decomposition rate and extensive surface disintegrations, respectively, indicating the surface bacterial colonization and degradation. Furthermore, this investigation also provided the details of physico-chemical nature of polymeric e-waste used in this study. Therefore, this is a sincere effort to resolve the anomaly between monoculture and consortium for eco-friendly management and bio-recycling of e-waste. Hence, it is proposed that monoculture *P. aeruginosa* strain PE10 can be used singly for large-scale biological management of e-waste sustainably in the near future.

## Data availability statement

The original contributions presented in the study are included in the article/Supplementary material, further inquiries can be directed to the corresponding author.

## Author contributions

PD: Data curation, Formal analysis, Investigation, Writing—original draft, Resources. DS: Formal analysis, Investigation, Methodology, Writing—review & editing. SK: Writing—review & editing, Software. MZ: Formal analysis, Data curation, Writing—review & editing. RG: Conceptualization, Resources, Supervision, Writing—review & editing, Validation.
