# Supplementary material for: Comparative e-waste plastics biodegradation efficacy of monoculture Pseudomonas aeruginosa strain PE10 and bacterial consortium under in situ condition
Source: Front Microbiol. 2024 Jan 18;14:1277186. doi: 10.3389/fmicb.2023.1277186 (PMC10830738; doi:10.3389/fmicb.2023.1277186)
Supplement: Supplementary file 1 [file Data_Sheet_1.doc]

**Supplementary Text 1.**

Shelf-life of strain PE10 and consortium in the talc based bioformulation was inferred by serial dilution plating method on NA plates. To check the viability, 1 g of bioformulation was added in 1 mL of sterilized distilled water and the suspension was further added in 9 ml of sterilized water. Likewise, serial dilution was made and the CFU/mL were counted initially after 2 and 4 days of incubation at 35°C. Next, the same step was followed to determine the viability after 7 days of regular interval following 21 days, trailed by 15 days interval. This study was assessed to verify the alterations in bioformulation through the calculation of CFU/mL at the time of storage under ambient room temperature.


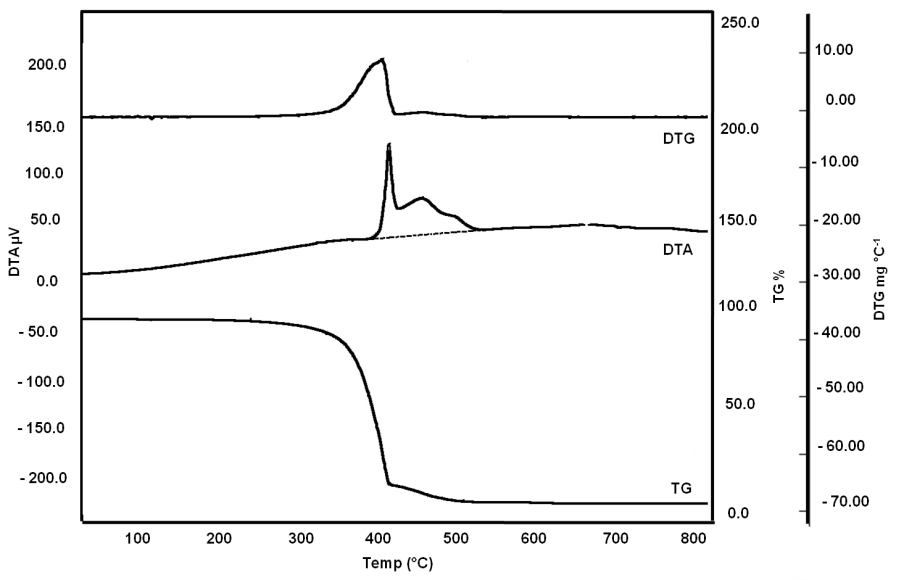

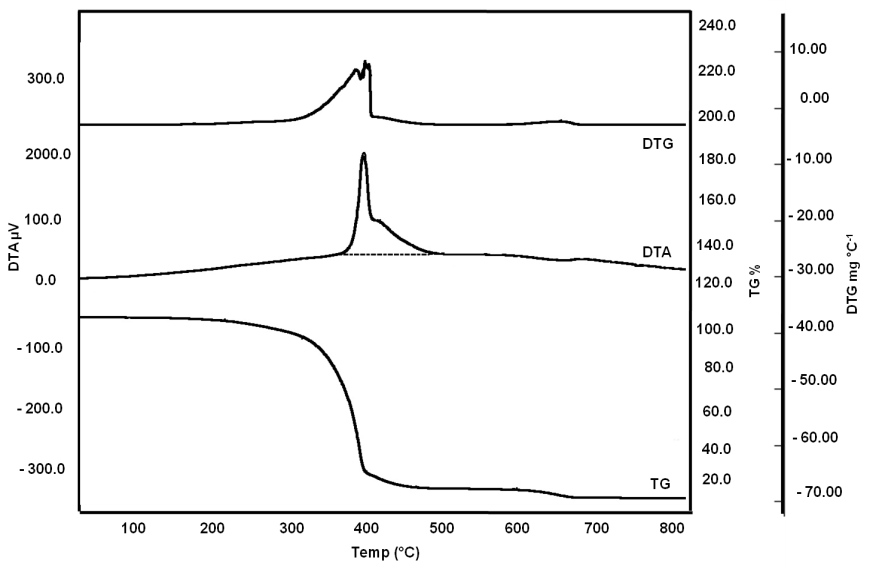


**(a) (b)**


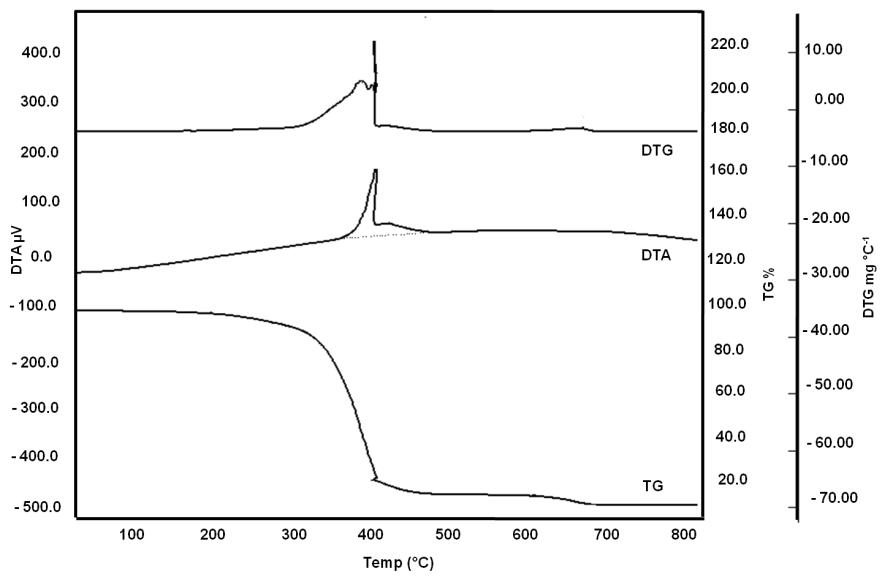


**(c)**

**Supplementary Fig. 1:** Comparative thermal analysis of biodegraded e-waste after 3 months of soil incubation, where (a) represent untreated control and (b) and (c) represents e-waste treated with bacterial consortium and strain PE10, respectively


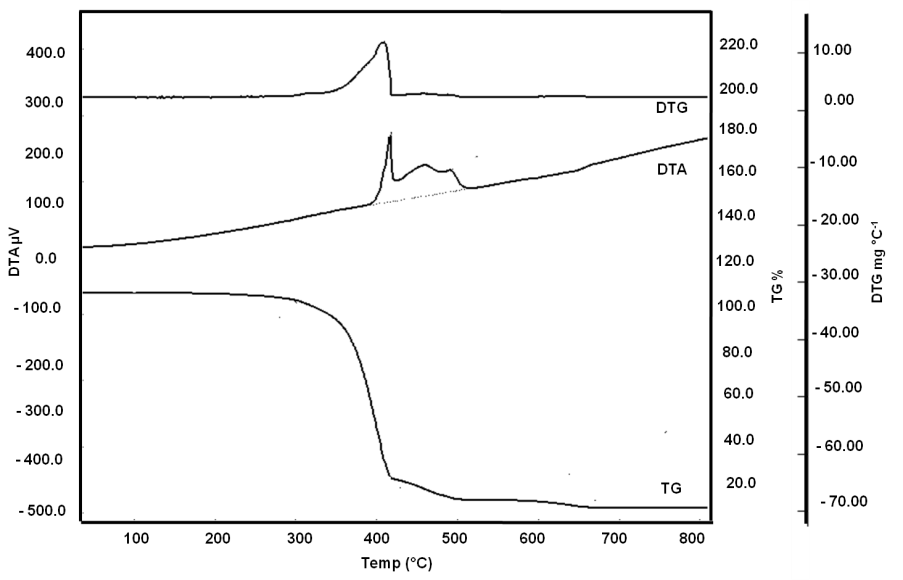

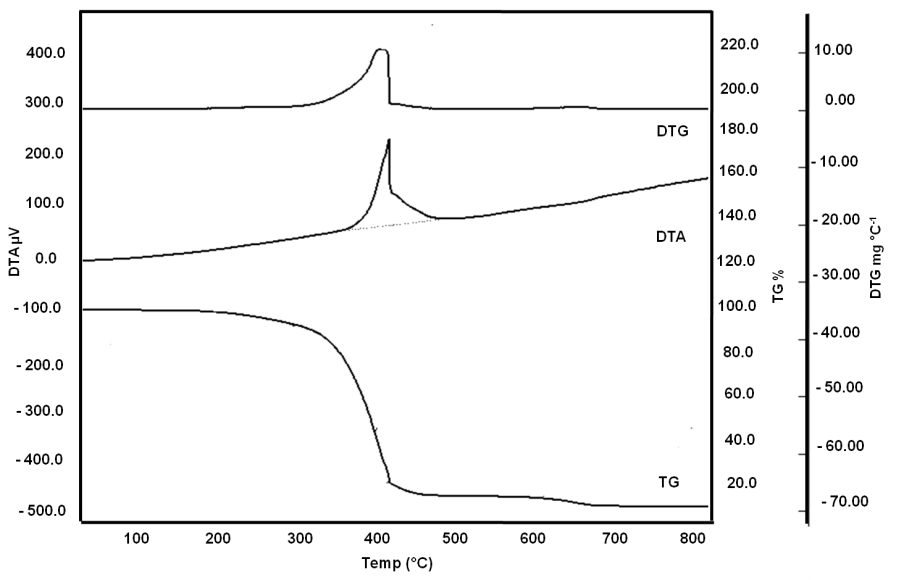


**(a) (b)**


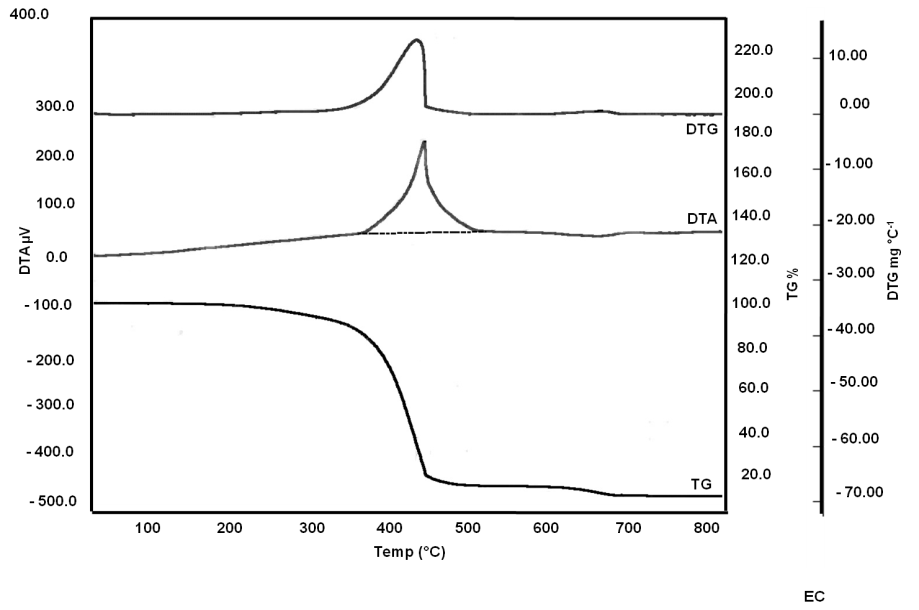


**(c)**

**Supplementary Fig. 2:** Comparative thermal analysis of biodegraded e-waste after 6 months of soil incubation, where (a) represent untreated control and (b) and (c) represents e-waste treated consortium and strain PE10, respectively

**Supplementary Table 1.** Characterization of pure e-waste used under study through FTIR absorptions

| **Samples** | **FTIR cm^-1^** | | | | | | | |
| --- | --- | --- | --- | --- | --- | --- | --- | --- |
|  | **ν O-H** | **ν C-H (asym)** | **δ C=O** | **ν C=C** | **δ C-H** | **ν C-O** | **ν C-O-C (sym)** | **ν C-Cl** |
| **Pure e-waste** | 3390.49 | 2922.8 | 1755.21 | 1645.87 | 1402.48 | 1155.8 | 1069.02 | 759.38 |

**ν-**Stretching, **δ-**Bending, **asym-**Asymmetric, **sym**-Symmetric

**Supplementary Table 2:** Comparative FTIR absorptions depicting progressive e-waste biodegradation under *in situ* conditions after treatment with respective consortium and monoculture strain PE10 for 3 months

| **Samples** | **FTIR cm^-1^** | | | | | | | | | | | | | |
| --- | --- | --- | --- | --- | --- | --- | --- | --- | --- | --- | --- | --- | --- | --- |
|  | **ν O-H** | **ν C-H (asym)** | **δ C=O** | **ν C=C** | **δ N-H** | **ν ring C=C** | **δ C-H** | **ν C-O-C (asym)** | **ν C-O** | **ν C-O-C (sym)** | **ν C-H (sym)** | **ν C-Cl** | **δ­­­ =C-H** | **δ­­­ ≡C-H** |
| **Untreated control** | 3391.65 | 2923.58 | 1730.09 | 1641.54 | **-** | - | 1386.95 | - | 1117.49 | ***** | **-** | 758.79 | **701.06** | - |
| **Consortium treated e-waste** | 3391.89 | ***** | ***** | 1647.62 | **-** | - | 1385.28 | - | ***** | ***** | **-** | ***** | - | **618.41** |
| **PE10 treated e-waste** | 3021.99-3371.40 | 2852.99-2924.87 | ***** | ***** | **1600.96** | **1450.99-1492.80** | ***** | **1215.11** | ***** | 1026.46 | **928.74** | 758.26 | **668.58-701.20** | - |

**ν-**Stretching, **δ-**Bending, **asym-**Asymmetric, **sym**-Symmetric, *****=Sign of deletion

**Supplementary Table 3:** Comparative FTIR spectral analysis of progressive e-waste biodegradation under *in situ* conditions after treatment with respective consortium and *P. aeruginosa* strain PE10, over incubation period of 6 months

| **Samples** | **FTIR cm^-1^** | | | | | | | | | | | | |
| --- | --- | --- | --- | --- | --- | --- | --- | --- | --- | --- | --- | --- | --- |
|  | **ν O-H** | **ν C-H (asym)** | **ν N-H** | **δ C=O** | **ν C=C** | **δ N-H** | **ν ring C=C** | **δ C-H** | **ν C-O-C (asym)** | **ν C-O** | **ν C-O-C (sym)** | **ν C-Cl** | **δ­­­ =C-H** |
| **Untreated control** | 3023.75 | 2925.22 | **-** | 1723.33-1771.17 | ***** | - | **1452.18-1502.65** | ***** | - | 1164.05-1195.66 | 1015.71 | 757.72 | **669.26-701.42** |
| **Consortium treated e-waste** | 3026.36 | 2923.76 | **-** | ***** | ***** | **1600.69** | **1451.4** | ***** | **1222.24** | ***** | ***** | 757.33 | **699.76** |
| **PE10 treated e-waste** | 3023.17 | 2852.67-2924.19 | **2402.35** | ***** | ***** | **1601.03** | **1451.97-1492.72** | ***** | **1216.43** | ***** | 1027.29 | 759.44 | **667.13-700.43** |

**ν-**Stretching, **δ-**Bending, **asym-**Asymmetric, **sym**-Symmetric, *****=Sign of deletion
